# High Reinforcing Efficacy of Nicotine in Non-Human Primates

**DOI:** 10.1371/journal.pone.0000230

**Published:** 2007-02-21

**Authors:** Bernard Le Foll, Carrie Wertheim, Steven R. Goldberg

**Affiliations:** 1 Preclinical Pharmacology Section, National Institute on Drug Abuse, National Institutes of Health-Department of Health and Human Services, Baltimore, Maryland, United States of America; 2 Translational Addiction Research Laboratory, Centre for Addiction and Mental Health and University of Toronto, Toronto, Canada; James Cook University, Australia

## Abstract

Although tobacco appears highly addictive in humans, there has been persistent controversy about the ability of its psychoactive ingredient nicotine to induce self-administration behavior in laboratory animals, bringing into question nicotine's role in reinforcing tobacco smoking. Because of ethical difficulties in inducing nicotine dependence in naïve human subjects, we explored reinforcing effects of nicotine in experimentally-naive non-human primates given access to nicotine for periods of time up to two years. Five squirrel monkeys with no experimental history were allowed to intravenously self-administer nicotine by pressing one of two levers. The number of presses on the active lever needed to obtain each injection was fixed (fixed-ratio schedule) or increased progressively with successive injections during the session (progressive-ratio schedule), allowing evaluation of both reinforcing and motivational effects of nicotine under conditions of increasing response cost. Over time, a progressive shift toward high rates of responding on the active lever, but not the inactive lever, developed. The monkeys' behavior was clearly directed toward nicotine self-administration, rather than presentation of environmental stimuli associated with nicotine injection. Both schedules of reinforcement revealed a high motivation to self-administer nicotine, with monkeys continuing to press the lever when up to 600 lever-presses were needed for each injection of nicotine. Thus, nicotine, by itself, in the absence of behavioral or drug-exposure history, is a robust and highly effective reinforcer of drug-taking behavior in a non-human primate model predictive of human behavior. This supports the use of nicotinic ligands for the treatment of smokers, and this novel preclinical model offers opportunities to test future medications for the treatment of nicotine dependence.

## Introduction

Tobacco dependence is described as a chronic, relapsing disorder in which compulsive drug-seeking and drug-taking behavior persists despite negative consequences and the motivation to quit. The highly addictive properties of tobacco are exemplified by the great difficulty in quitting smoking. Although most smokers express a desire to stop smoking, only a small percentage of subjects succeed [1], [2]. Surprisingly, reinforcing effects of nicotine alone have often been difficult to demonstrate directly in controlled laboratory studies with both animals and humans as experimental subjects. Consequently, there has been continuing controversy in the literature about the validity of previous findings of reinforcing effects of nicotine in experimental animals and human subjects [3], [4], [5], [6], [7], [8].

The first attempts to demonstrate nicotine self-administration in drug-naive animals were performed in non-human primates. Table 1 provides an overview of the published studies performed with the nicotine self-administration paradigm in non-human primates. Most of these studies do not support the conclusion that nicotine can function as an effective reinforcing agent. Without specific conditions, such as automatic nicotine infusions, previous self-administration of other drugs or food, or food-deprivation, nicotine previously failed to maintain significant self-administration behavior in non-human primates (Table 1). These findings are in striking contrast to the vast literature that indicates that other drugs of abuse such as cocaine or opiates can initiate and maintain self-administration behavior in non-human primates. However, these studies with nicotine self-administration in non-human primates used experimental conditions, such as very slow injection duration or pre-training on cocaine, that may not have been adequate.

**Table 1:** Summary of previous studies evaluating intravenous nicotine self-administration behavior in non-human primates.

Species	Schedule	Previous training and exposure to experimenter administered non-contingent nicotine injections	Nicotine self-administration	Ref
Rhesus	FR	Absence of automatic nicotine injections	No	[57]
		Presence of automatic nicotine injections	Yes	
Rhesus	FR or PR	History of self-administering other drugs	No	[58]
Baboons	FR	Food or cocaine self-administration history	No	[39]
Squirrel	Second-order	Priming injection and cocaine self-administration history	Yes	[14]
Squirrel	Fixed-interval	Automatic injections or cocaine self-administration history	Yes	[41]
Baboons	FR	Drug self-administration history (for 3 out of 4 monkeys)	No	[40]
Rhesus	FR	Food-restriction and previous operant training	No	[59]
Rhesus	FR	Cocaine self-administration history	No	[60]
Squirrel	FR	Cocaine self-administration history	Yes	[61]
Rhesus	FR	Drug self-administration history	Yes	[42]

The experimental conditions used may strongly influence the outcome of drug self-administration studies in rodents. Indeed, without adequate conditions, the initial explorations performed in rats suggested that nicotine did not possess any reinforcing properties [5]. Although, some investigators have been able to obtain significant intravenous self-administration behavior with nicotine in rats [9], the findings of these studies have often not been reproduced [10] and the conclusions have been critized due to the fact that most studies employed conditions such as priming injections of nicotine before sessions, food deprivation or previous food self-administration training, that may have resulted in non-specific responding [5]. The difficulty in obtaining significant self-administration suggests that the reinforcing effects of nicotine are weak in rodents. This hypothesis is supported by the limited efficacy of nicotine to induce significant conditioned place preferences (see [11] for a review) and is in opposition to the apparent high reinforcing effects of tobacco in human smokers.

This discrepancy between the apparent high reinforcing effects of tobacco in humans and the apparent weak reinforcing effects of nicotine in experimental animals has led to numerous theories, the most recent ones being that nicotine may produce its reinforcing effects indirectly through its conditioning properties [12] or that other substances contained in tobacco smoke may be necessary for the reinforcing effects of tobacco [10], [13].

The critical role environmental stimuli play in the maintenance of intravenous nicotine self-administration behavior was initially proposed following the description of the ability of nicotine to maintain responding under a second-order schedule of intravenous nicotine self-administration in squirrel monkeys [14]. With this schedule of reinforcement, responding is maintained to a large extent by the brief light stimuli which are only intermittently paired with nicotine delivery. This hypothesis is supported by recent rodent experiments. In these experiments, light stimuli were paired with each self-administered injection of nicotine. In one study with rats, discontinuing presentation of the environmental stimuli paired with intravenous nicotine injection decreased self-administration behavior almost as effectively as the removal of nicotine itself [12], [15], [16]. In another recent study with rats, response-contingent presentation of stimuli previously paired with nicotine injections, by themselves, continued to maintain levels of responding equal to those previously maintained by injections of nicotine for up to three months, demonstrating the persistent nature and high motivational value of these environmental stimuli [17].

One theory that has been recently proposed to explain the difficulties involved in obtaining nicotine self-administration is that substances other than nicotine in tobacco smoke possess psychoactive effects and contribute to the reinforcing effects of tobacco smoking [18]. Among the likely candidates are substances present in tobacco smoke that are able to inhibit monoamine-oxidase (MAO) enzymes, which are inhibited in the brain of smokers [19], [20]. Recent results obtained in rats suggest that treatment with MAO inhibitors may potentiate the reinforcing effects of intravenously self-administered nicotine [21]. However, conflicting results have been obtained in mice [22] and the results obtained in rats were obtained with a degree of MAO inhibition that is much higher than that observed in the brains of smokers.

Another possibility that may explain the discrepancy between the apparent high reinforcing effects of tobacco in humans and the apparent weak reinforcing effects of nicotine in rodents is that nicotine may possess higher reinforcing effects in primates than in rodents and that these reinforcing effects have not been revealed by previous investigations in primates due to inappropriate conditions. Here we explored this hypothesis by evaluating the direct reinforcing effects of nicotine in experimentally-naive squirrel monkeys using a fixed-ratio (FR) schedule of reinforcement. The squirrel monkeys studied were experimentally naive at the beginning of the study: they had no history of exposure to drugs other than anesthetics used for surgery and antibiotics, no history of drug self-administration and had not been trained to respond for food. Due to the growing literature obtained in rodents suggesting that nicotine may act by increasing the motivational value of environmental stimuli associated with its effects, brief light stimuli were associated with each completion of the FR response requirement on both active and inactive levers. Responding for various doses of nicotine was evaluated and dose-response curves generated once the monkeys learned to quickly reduce their responding when saline was substituted for nicotine. Nicotine self-administration responding of the monkeys was also evaluated under a progressive-ratio (PR) schedule of reinforcement which provided a motivational measure of the amount of effort monkeys would exert to obtain an intravenous injection of nicotine.

## Results

### Acquisition of nicotine self-administration behavior

**Figure 1:**
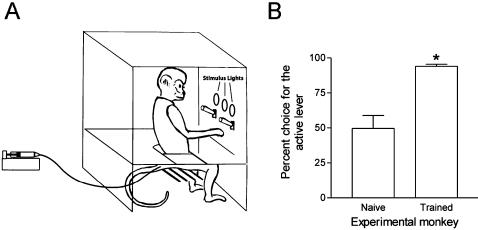
A. Monkeys sat in chambers equipped with two levers and distinctly colored light stimuli above the levers. Completion of the response requirement (the ratio) on the active lever produced a brief two-sec presentation of a light stimulus and an intravenous injection of nicotine followed by a timeout (TO) period of 5 to 60 sec. Completion of the ratio requirement on the inactive lever resulted in presentation of a brief two-sec light stimulus of a different color but no injection. The fixed-ratio (FR) response requirement was gradually increased over successive sessions from one to ten (FR 1 to FR 10). B. Mean percentage choice for responding on the active lever by monkeys when they were experimentally naive (first week under a FR 1 schedule) and when they had learned to self-administer nicotine under the FR 10, TO 60 sec schedule (first week under the FR 10 schedule). *P<0.01, compared to first week of training.

All the monkeys studied acquired nicotine self-administration under the FR schedule of reinforcement. The monkeys were initially allowed to self-administer 10 µg/kg nicotine injections under a FR 1 schedule of reinforcement and, over subsequent sessions, the response requirement was gradually increased to FR 10. It took 25 to 101 sessions (mean 70±17 sessions) for monkeys to reach the final FR 10, time-out (TO) 60 sec schedule. During the first week of acquisition, no preference was noted for the active versus the inactive lever (percentage choice on the active lever was 49.6±9.3%, as expected by chance) (Fig. 1B). This indicates that during these initial sessions, the animals produced a similar number of two-sec light presentations by responding on the active and the inactive levers. However, over repeated sessions the monkeys developed a strong preference for responding on the active-lever compared to the inactive-lever (P<0.01) and responding on the inactive-lever dropped to negligeable levels (Fig. 1B).

### Extinction of nicotine self-administration behavior

**Figure 2:**
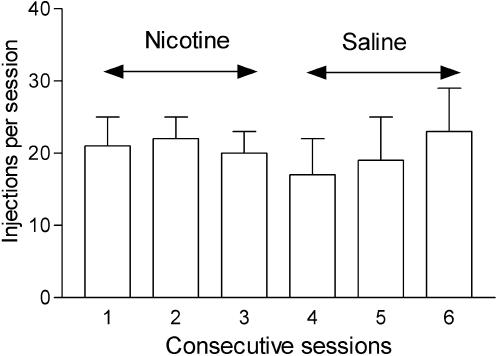
Maintenance of self-administration behavior under the FR 10 schedule during the first experience with saline substitution. Mean number (±SEM) of ratios completed on the active lever during three consecutive session with access to nicotine followed by an additional three sessions with saline substituted for nicotine are shown. The brief 2-sec light stimuli were presented following each ratio completion during both the nicotine and saline sessions. Self-administration behavior was not reduced by the substitution of saline injections for nicotine injections during this first exposure to extinction conditions.

The first time saline was substituted for nicotine under the FR 10 schedule, responding on the active lever continued undiminished, suggesting that the nicotine-associated brief light stimuli previously associated with nicotine injection, were as effective as nicotine itself in maintaining self-administration responding. Analysis of the number of injections per session over six consecutive sessions, three with nicotine injections followed by three with saline substituted for nicotine, revealed that substitution of saline for nicotine initially had no effect on responding (F4,20 = 0.22, P = 0.9, Fig 2).

**Figure 3:**
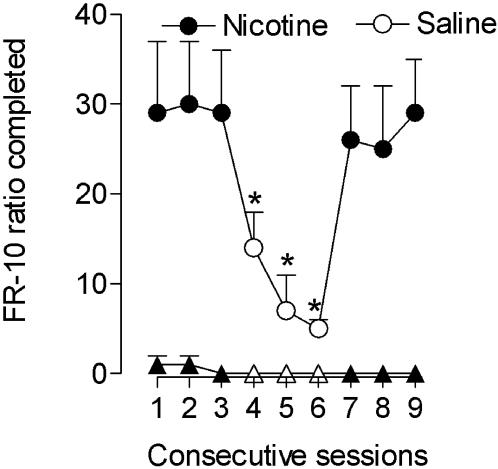
Maintenance, extinction and reacquisition of self-administration behavior over consecutive sessions under the FR 10 schedule of reinforcement. Numbers of injections per session during consecutive nicotine (10 µg/kg per injection, filled symbols) and saline self-administration sessions (open symbols) are presented. *Symbols* represent the mean (±SEM) number of ratios completed on the active (circle) or inactive (triangle) levers per session from five squirrel monkeys. *P<0.05, compared to nicotine sessions.

**Figure 4:**
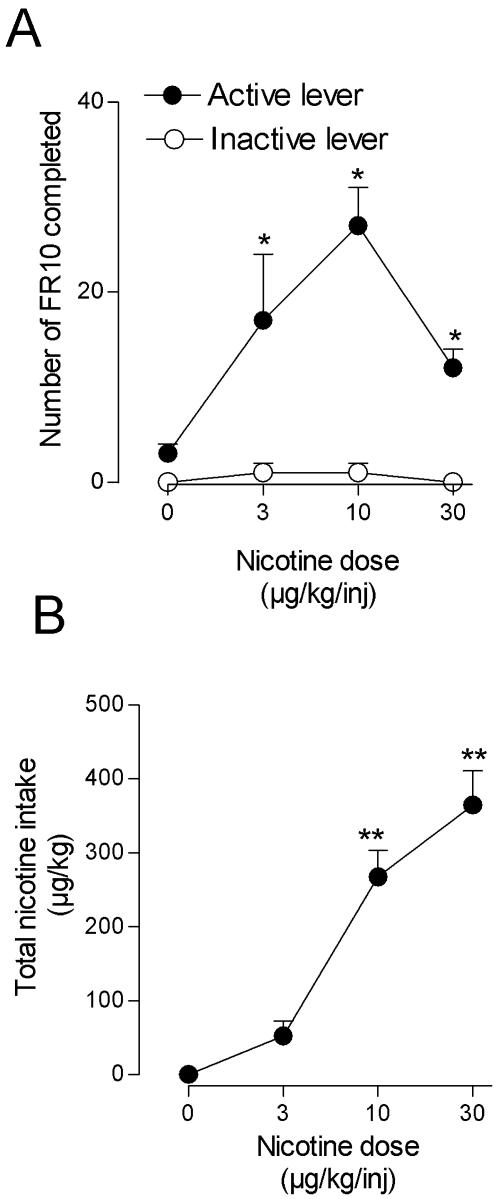
Influence of nicotine dose on nicotine self-administration and total nicotine intake per session under the FR 10 schedule. Number of fixed ratios completed on the active and inactive levers per session (A) and total nicotine intake per session (B) are presented as a function of injection dose of nicotine (n = 5). Each *symbol* represents the mean (±SEM) of at least three sessions under each nicotine injection dose condition *P<0.05, **P<0.01 post-hoc comparisons with the saline vehicle (0 µg/kg per injection) conditions.

After acquisition of stable nicotine self-administration behavior under the FR 10, TO 60 sec schedule, the monkeys again had saline substituted for nicotine over repeated sessions. Responding of three of the monkeys extinguished after 5 to 17 sessions of saline substitution. Two monkeys did not initially show extinction of responding under the FR 10 schedule and were switched to the progressive-ratio schedule. These two monkeys showed extinction of responding after 7 or 13 sessions of responding for saline under the progressive-ratio schedule and were then returned to the FR 10 schedule. After this history, the number of injections self-administered per session immediately decreased to low levels when saline injections were again substituted for 10 µg/kg nicotine injections under the FR 10 schedule and self-administration responding immediately returned to high levels when saline injections were replaced with 10 µg/kg nicotine injections (Fig. 3). Repeated measures ANOVA indicated a significant effect of lever (*F1*,64 = 19.9, *P* = 0.002), a significant effect of time (*F*8,64 = 7.4, *P*<0.0001), and a significant time x lever interaction (*F*8,64 = 7.4, *P*<0.0001). Post-hoc analysis indicated that the number of ratios completed on the active lever were significantly lower during saline substitution compared to nicotine acess conditions (all P<0.001). In contrast, no significant changes were noted in the number of ratios completed on the inactive lever (all P>0.95). Under the final FR 10 TO 60 sec schedule, injections of 10 µg/kg nicotine maintained relatively high response rates (0.15±0.04 response/s compared to 0.01±0.001 response/s for saline. P<0.01) and about 50% of the maximal possible number of injections per session was self-administered each session (27±4 injections/1-hour session) (Fig. 4A).

### Nicotine dose variations under the fixed-ratio schedule

Varying the nicotine dose per injection resulted in an inverted U-shaped dose-effect curve, similar to curves typically obtained with other abused drugs under FR schedules of intravenous drug self-administration (Fig 4A). Two-way analysis ANOVA indicated that there was a significant effect of lever choice (active vs inactive, *F*1,32 = 46.5, *P*<0.0001), a significant effect of nicotine dose (*F*3,32 = 6.3, *P = 0.002*) and a significant interaction between lever choice and nicotine dose (*F*3,32 = 5.4, *P = 0.004*). Post-hoc analysis indicated that all nicotine doses maintained high rates of responding on the active lever compared to saline substitution conditions (P = 0.002, P<0.0001 and P = 0.04 for nicotine 3, 10, 30 µg/kg per injection, respectively). There was no effect of nicotine dose on responding on the inactive lever (all P>0.4). There was a significant effect of nicotine dose on total nicotine intake during the 1-hour session (*F3*,16 = 40.4, *P*<0.0001) (Fig. 4B). Post-hoc analysis indicated that total nicotine intake significantly increased when the monkey had access to 10 µg/kg and 30 µg/kg nicotine injections (all P<0.0001 compared to saline acess conditions). Patterns of responding by the monkeys were typical of FR responding maintained by other drugs of abuse: when the green light was illuminated, an initial pause in responding was followed by an abrupt change to rapid responding that continued until the ratio was comleted. Rates of responding were very low during saline substitution and were highest at the peak dose of 10 µg/kg per injection of nicotine.

### Nicotine self administration under the progressive-ratio schedule

**Figure 5:**
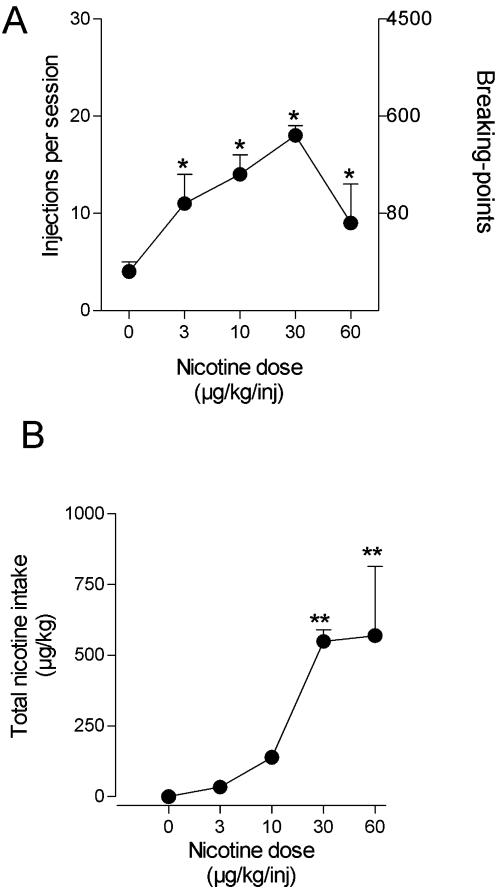
Influence of nicotine dose on nicotine self-administration and total nicotine intake per session under the progressive-ratio schedule. Number of nicotine injections per session and corresponding breaking-point values (highest ratio completed) under the progressive-ratio schedule (A) and total nicotine intake per session (B) are presented as a function of injection dose of nicotine (n = 5). Each *symbol* represents the mean (±SEM) of at least three sessions under each nicotine injection dose condition *P<0.05, **P<0.01 post-hoc comparisons with saline vehicle (0 µg/kg per injection) conditions.

Under the progressive-ratio schedule, responding seldom occurred on the inactive lever and almost all ratios completed by the monkeys were on the active lever, in agreement with the strong preference for the active lever that had developed over time under the FR schedules (see above). There was a significant effect of nicotine dose on total number of injections during the session (F4,20 = 6.9, P = 0.001) and on total nicotine intake (F4,20 = 10.8, P<0.0001). Post-hoc analysis indicated that all nicotine doses maintained high rates of responding on the active lever compared to rates of responding during saline substitution (P = 0.03, P = 0.002, P<0.0001, P = 0.04 for nicotine 3, 10, 30 and 60 µg/kg per injection, respectively) . Again, there was an inverted U-shaped dose-effect curve, with the 30 µg/kg per injection dose of nicotine maintaining peak values for number of injections per session and breaking point (Fig. 5A) and a near maximal nicotine intake per session (Fig. 5B). When nicotine dose was further increased to 60 µg/kg per injection, the number of injections per session and breaking point values decreased (Fig. 5A) and there was no further increase in nicotine intake per session (Fig. 5B). Under the progressive-ratio schedule, monkeys continued to press the lever when up to 600 lever-presses were needed for a single nicotine injection, clearly demonstrating a high motivation to self-administer nicotine (Fig. 5A).

## Discussion

The present findings provide a clear demonstration of nicotine's effectiveness as a reinforcer of drug-taking behavior in experimentally naive non-human primates. In the present experiments, nicotine initiated and sustained very high rates of intravenous self-administration behavior in squirrel monkeys without a history of exposure to other drugs or behavioral training and without any facilitory conditions, such as food-deprivation or non-contingent automatic injections of nicotine before or during experimental sessions. There was a high motivation to obtain nicotine under both the FR and the progressive-ratio schedules. Under the FR schedule, about 50% of the maximal possible number of injections per session was self-administered each session and, under the progressive-ratio schedule, monkeys continued to press the lever at a high rate when up to 600 lever-presses were needed for each injection of nicotine.

Under the present FR schedule of intravenous drug self-administration, the pattern of responding maintained by nicotine was similar to patterns of responding maintained by intravenous injections of cocaine, *d*-amphetamine, methohexital or delta-9-tetrahydrocannabinol (THC) in previous studies using the same primate species and similar FR schedules of intravenous drug injection [23], [24], [25], [26] although the present peak rate of responding with nicotine was less intense than peak rates of responding previously reported with other drugs of abuse such as cocaine, d-amphetamine and THC. This is in agreement with results from a previous study with rats, which directly compared, in the same animals, the reinforcing effects of nicotine with those of cocaine under a progressive-ratio schedule and found that the reinforcing effects of nicotine were weaker than those of cocaine [27] and that animals prefered cocaine over nicotine, when given access to both drugs during the same session [27].

This is the first report of clear reinforcing effects of nicotine in non-human primates using the progressive-ratio schedule of reinforcement and the first reported use of this schedule of reinforcement with squirrel monkeys. Breaking-point values were relatively high in the squirrel monkeys (around 600 lever press to get a single nicotine injection), suggesting that nicotine possesses high motivational effects in primates. It is interesting that the present levels of responding maintained by intravenous nicotine in squirrel monkeys are relatively close to levels of responding recently reported in human smokers self-administering nicotine intravenously [28]. In the human study, when the number of responses required for each injection was progressively increased over consecutive sessions, high rates of responding were maintained by nicotine injections, but not by concurrently available saline injections, at response requirements as high as 1600 responses per injection. Mean rates of responding were between 2.3 and 3.3 responses per second for nicotine injections at maximal FR values ranging from 400 to 1600 in individual subjects, compared to under 0.3 responses per second for concurrently available saline placebo injections [28]. In contrast to the present findings with squirrel monkeys and and our previous findings with human subjects, levels of responding obtained with rodents in nicotine self-administration studies have been significantly less intense under progressive-ratio schedules, with breaking-points values between 25 and 100 depending on the dose of nicotine used and the investigators [29], [30], [31], [32], [33]. Also, the number of nicotine injections per session is generally much lower in rats compared to squirrel monkeys or humans, with the average number of injections per session ranging between 6 to 10 injections per session in most studies [29], [30], [31], [32], [33].

In the present study, environmental stimuli (colored lights) were paired with each nicotine injection during acquisition of the self-administration behavior. This appeared to contribute to the ability of nicotine to act as a reinforcer, in agreement with several recent rodent experiments. The finding that self-administration responding continued to be maintained at high rates during the first exposure to saline substitution conditions (Fig. 2) suggests that habitual behavior had progressively developed in the monkeys and that they were becoming insensitive to the outcome of their actions. Another likely hypothesis, however, is that self-administration behavior during the initial saline substitution sessions was maintained by light stimuli that had been previously associated with nicotine injections and continued to be presented in association with saline injections [17], [34].

Nicotine, like other psychostimulant drugs [35], also produces unconditioned effects, unrelated to reinforcement, that can increase the efectiveness of non-drug environmental stimuli as reinforcers and this process can occur independently of any direct temporal association between nicotine administration and stimulus presentation [15], [36], [37]. In our experiments, however, during the first week of acquisition of nicotine self-administration behavior, the monkeys had similar exposure to brief light stimuli associated with responding on both the active and inactive levers, since responding on both levers was identical (Fig 1), but there was a progressive shift in subsequent weeks toward responding on the active lever. This progressive shift towards increased responding on the active lever and reduced responding on the inactive lever, which occurred even though responding on the inactive lever continued to produce brief light stimuli, suggests that responding of experienced animals was directed more toward delivery of nicotine than toward presentation of the associated brief light stimuli. It is also important to note that there was nicotine exposure throughout each session as a result of responding on the active lever and that exposure to nicotine alone did not result in the brief light presentations produced by responding on the inactive lever acquiring reinforcing efficacy. Finally, after repeated exposure to cycles of saline substitution, substitution of saline for nicotine did result in decreases in self-administration responding. Thus, in these experienced monkeys, nicotine-associated stimuli, by themselves, appeared unable to maintain significant self-administration behavior under fixed- and progressive-ratio schedules.

The present findings contrast with the previous difficulties in obtaining significant nicotine self-administration in non-human primates (Table 1) and rodents. Several factors may explain such discrepancies. An analysis of the speed of injection used in the previous studies suggests that a rapid speed of nicotine injection, which mimic kinetics of nicotine delivery with tobacco smoking [38] may have been critical. Previous studies that used very slow (2-min) injections of nicotine [39] or slow (5 sec or more) injections of nicotine [40] did not provide significant evidence for reinforcing effects of nicotine, whereas, nicotine had significant reinforcing effects when injection durations of less than one sec were used [14], [41]. The importance of injection speed is also supported by findings from one study that directly measured rates of nicotine self-administration at different injection durations [42]. However, injection speed cannot explain the lower reinforcing effects of nicotine in rodents, since most of the rodent studies employed relatively high infusion speeds.

Rodent self-administration studies generally last a maximum of three to four months, due to catheter loss. In contrast, our monkey self-administration studies extended over periods of one to two years and this may have allowed time for monkeys to become tolerant to some aversive effects of nicotine, thus revealing nicotine's high reinforcing effects. It has long been known that nicotine can produce both reinforcing and aversive effects, sometimes at the same dose, depending on the experimental schedule under which nicotine is made available and the subject's history [43], [44]. Humans report both positive and negative effects following nicotine injections [28], [45], [46], [47]. In agreement, the same dose of nicotine may produce either positive or aversive motivational effects in rats using conditioned place preference procedures [11], [48] and in squirrel monkeys using self-administration and punishment procedures [14], [18], [44]. Although we cannot directly evaluate this hypothesis, it is possible that prolonged exposure to nicotine over many months may have allowed the monkeys to develop tolerance to some aversive effects of nicotine and, therefore allowed the high reinforcing effects of nicotine to develop.

Finally, the monkeys in this study had no previous experience with self-administration of other psychoactive drugs with different pharmacological effects (e.g. cocaine), that may have influenced effects of nicotine in earlier studies using non-human primates [49]. Exposure to one class of drugs may alter the subsequent effects of drugs from another pharmacological class [49], [50], [51]. For example, aversive effects of high doses of THC are revealed in nicotine-experienced rats [51] and lower rates of intravenous THC self-administration are described in monkeys with a history of cocaine self-administration [25] compared to drug-naive monkeys [26].

In conclusion, these findings provide a clear demonstration that nicotine, by itself, in the absence of a behavioral-testing or drug-exposure history or existing conditions, such as food deprivation or experimenter-administered nicotine injections, is a robust and highly effective reinforcer of drug-taking behavior in a non-human primate model predictive of human behavior. The monkeys' behavior was clearly directed toward self-administration of nicotine, rather than presentation of environmental stimuli associated with nicotine's effects. The experiments conducted in monkeys without an extinction history also confirm the critical influence of nicotine associated-stimuli in sustaining self-administration behavior. Nicotine-seeking was persistent and robust, even in the face of increases in response cost per nicotine dose (price) by a factor of sixty. These findings support the use of nicotinic ligands for the treatment of tobacco dependence. Moreover, the self-administration of nicotine by squirrel monkeys provides a reliable animal model of nicotine dependence, suitable for developing new therapeutic strategies for the treatment or tobacco smoking in humans. This model can be used to validate the use of novel therapeutic approaches such as dopamine D_3_ ligands [52], [53] or cannabinoid CB_1_ ligands [54], [55].

## Material and Methods

### Subjects

Five naive male squirrel monkeys (Saimiri sciurea), weighing 730 to 950 g were subjects. They were housed individually in a temperature- and humidity-controlled room and were maintained on a 12-h light/dark cycle; the lights were on from 6:45 AM to 6:45 PM. Experiments were conducted during the light phase. Animals were maintained in facilities fully accredited by the American Association for the Accreditation of Laboratory Animals used in this study were maintained in facilities fully accredited by the American Association for the Accreditation of Laboratory Animal Care (AAALAC) and all experimentation was conducted in accordance with the guidelines of the Institutional Care and Use Committee of the Intramural Research Program, National Institute on Drug Abuse, National Institutes of Health, and the Guide for Care and Use of Laboratory Animals (National Research Council 2003). In each monkey, a polyvinyl chloride catheter (inside diameter, 0.38 mm; outside diameter 0.76 mm) was used for i.v. injection of drug and was passed through the right or left jugular vein or through the femoral vein to the level of the right atrium under halothane anesthesia. Subcutaneous catheters led to the monkey's back where they exited the skin. The monkeys wore jackets at all times to protect these catheters. Each weekday the catheters were flushed, refilled with saline (0.9% NaC1) and sealed with stainless steel obturators. Before acquisition of nicotine self-administration, the monkeys have been euthanized to performed brain imaging experiment using positron emission tomography to measure the expression of brain nicotinic receptors. These results will be reported elsewere.

### Apparatus

During experimental sessions, monkeys sat in a Plexiglas chair similar to one previously described [25], [26] and were restrained in the seated position by a waist lock (Fig. 1A). The chair was enclosed in a sound-attenuating isolation chamber (model AC-3, Industrial Acoustics Co., Bronx, NY). Extraneous sounds were further masked by continuous white noise. Two-response levers were mounted in front of the monkeys. When the monkey pressed either lever with a force of 28 g or more, there was an audible relay click and a response was recorded. Three stimulus lights (green, amber and white) were mounted at eye level in front of the monkeys and used as visual stimuli (Fig. 1A). The middle green light was illuminated at the beginning of each session and turned off at the end of the session. The amber and white lights were located above the active and inactive levers, respectively, and were illuminated for 2 sec when a ratio was completed on the active or inactive lever. Teflon tubing connected the monkey's venous catheter to a syringe located outside the isolation chamber. The syringe was driven by a 110-volt a.c. motor, controlled by Med Associates (St. Albans, VT,USA) automatic programming equipment. Between experimental sessions, monkeys were kept in individual home cages with food and water freely available. No food-restriction was used.

### Nicotine self-administration

Initially, the monkeys were adapted to sitting in the chambers during repeated sessions. Then, daily experimental sessions (Monday through Friday) were started with each monkey. The green stimulus light was turned on at the beginning of each session and off at the end of the session. The duration of sessions was rapidly increased from 15 min to 60 min in less than two weeks. Initially only one response was required to obtain each 10 µg/kg intravenous injection of nicotine (one-response fixed-ratio schedule of reinforcement; FR 1) and after each injection the green light was turned off for 5 sec (5-sec time-out; TO 5 sec). Then, over subsequent sessions, the ratio requirement was progressively increased to a final value of FR10 and the TO duration was progressively increased to 60 sec. During each 60-min session, each completion of the ratio on the active lever resulted in illumination of the amber stimulus lights for two sec paired with an i.v. injection of 10 µg/kg of nicotine, followed by a 60-sec TO, during which the experimental chamber was dark and lever-press responses had no programmed consequences. Each completion of the ratio on the inactive lever resulted in illumination of the white stimulus lights for two sec but had no other programmed consequences.

### Nicotine self administration under the fixed-ratio schedule

Once the animal learned to quickly decrease its responding during sessions when saline was substituted for nicotine (extinction), the dose of nicotine maintaining responding was varied. Doses of 3, 10, 30 and 60 µg/kg of nicotine were studied. Each dose was evaluated for at least five consecutive sessions and the results of the last three sessions were usually used for analysis. Saline extinction sessions were conducted between testing of different nicotine doses.

### Nicotine self administration under the progressive-ratio schedule

After acquiring nicotine self-administration behavior under the FR schedule, monkeys were switched to the progressive-ratio schedule. Under the progressive-ratio schedule of intravenous nicotine injection, the response requirement increased with each successive injection. The steps of the exponential progression were adapted from those used by Roberts and Bennett [56], based on the following equation: response ratio = [5eX^(0.2xinfusion number)^]−5, rounded to the nearest integer. We chose this progression because it allows a more precise evaluation of the maximum response requirement that continued to maintain responding (the breaking-point value), than simply doubling the ratio requirement at each step. Since the monkeys first learned to self-administer nicotine under a FR 10 schedule, we used the closest value to 10 in this progression (i.e. 9) as the first step in the progressive ratio. Thus, the values of the steps were 9, 12, 15, 20, 25, 32, 40, 50, 62, 77, 95, 118, 145, 178, 219, 268, 328, 402, 492, 603, 737, 901 and 1102. Sessions under the progressive-ratio schedule lasted 6 hours or until 30 min passed without a response. The breaking point was defined as the step value of the last ratio completed before 30 min of non-responding or at the end of the 6 hours. Injection dose of nicotine was then varied. Doses of 0 (saline), 3, 10, 30 and 100 µg/kg/injection of nicotine were studied under the progressive-ratio schedule and each dose was studied for at least five consecutive sessions. Extinction studies were conducted between testing of different nicotine doses.

### Drugs

Nicotine [(-)-nicotine hydrogen tartrate] was purchased from Sigma Chemical Company (St Louis, Mo., USA). Nicotine was diluted in saline and the pH of nicotine solution was adjusted to 7.0 with dilute NaOH. Nicotine was administered intravenously using a solution containing 150 µg/kg nicotine/ml. We used a high speed of injection (200 msec), previously shown to facilitate nicotine self-administration and the volume of each injection was 0.2 ml for the 10 µg/kg/injection dose of nicotine. The duration and volume of injection were changed to vary dose of nicotine. No priming injections of nicotine were given before sessions and no non-contingent automatic injections of nicotine were given during sessions. Doses of nicotine were expressed as mg of free base per kg body weight.

### Data Analysis

Number of lever presses on active and inactive levers, and number of injections per sessions were recorded. The total nicotine dose received by the monkeys during the session was also calculated. The percentage of responses on the nicotine-associated active lever was calculated for the first five sessions of acquisition under the FR 1 schedule and for five consecutive sessions once stable nicotine self-administration was acquired. Choice of the active lever was calculated as a percentage of the number of responses made on the active lever relative to the total number of responses made on both levers during the session. Response rates were calculated for each session by dividing total responses in the presence of the green light by total time the green light was illuminated. Statistics were performed using either one-way or two-way ANOVA with nicotine dose, time or the lever as factors. LSD-post hoc tests were used.
